# Is There a Role for Base Excision Repair in Estrogen/Estrogen Receptor-Driven Breast Cancers?

**DOI:** 10.1089/ars.2014.6077

**Published:** 2014-12-01

**Authors:** Tarek M.A. Abdel-Fatah, Christina Perry, Arvind Arora, Nicola Thompson, Rachel Doherty, Paul M. Moseley, Andrew R. Green, Stephen Y.T. Chan, Ian O. Ellis, Srinivasan Madhusudan

**Affiliations:** ^1^ Department of Oncology, Nottingham University Hospitals, Nottingham, United Kingdom.; ^2^ Academic Unit of Oncology, Division of Cancer and Stem Cells, School of Medicine, University of Nottingham, Nottingham, United Kingdom.; ^3^ Division of Cancer and Stem Cells, Department of Pathology, School of Medicine, University of Nottingham, Nottingham, United Kingdom.

## Abstract

Estrogen and estrogen metabolite-induced reactive oxygen species generation can promote oxidative DNA base damage. If unrepaired, base damaging lesions could accelerate mutagenesis, leading to a “mutator phenotype” characterized by aggressive behavior in estrogen-estrogen receptor (ER)-driven breast cancer. To test this hypothesis, we investigated 1406 ER^+^ early-stage breast cancers with 20 years' long-term clinical follow-up data for DNA polymerase β (pol β), flap endonuclease 1 (FEN1), AP endonuclease 1 (APE1), X-ray cross-complementation group 1 protein (XRCC1), single-strand monofunctional uracil glycosylase-1 (SMUG1), poly (ADP-ribose) polymerase 1 (PARP1), ataxia telangiectasia mutated and Rad3 related (ATR), ataxia telangiectasia mutated (ATM), DNA-dependent protein kinase catalytic subunit (DNA-PKcs), Chk1, Chk2, p53, breast cancer susceptibility gene 1 (BRCA1), and topoisomerase 2 (TOPO2) expression. Multivariate Cox proportional hazards model was used to calculate a DNA repair prognostic index and correlated to clinicopathological variables and survival outcomes. Key base excision repair (BER) proteins, including XRCC1, APE1, SMUG1, and FEN1, were independently associated with poor breast cancer-specific survival (BCSS) (*p*s≤0.01). Multivariate Cox model stratified patients into four distinct prognostic sub-groups with worsening BCSS (*p*s<0.01). In addition, compared with prognostic sub-group 1, sub-groups 2, 3, and 4 manifest increasing tumor size, grade, mitosis, pleomorphism, differentiation, lymphovascular invasion, high Ki67, loss of Bcl-2, luminal B phenotype (*p*s≤0.01), and poor survival, including in patients who received tamoxifen adjuvant therapy (*p*<0.00001). Our observation supports the hypothesis that BER-directed stratification could inform appropriate therapies in estrogen-ER-driven breast cancers. *Antioxid. Redox Signal.* 21, 2262–2268.

## Introduction

Chronic estrogen exposure increases the risk of breast cancer (9). Although estrogen/estrogen receptor α (ERα)-dependent cellular proliferation signaling is widely known to promote breast cancer development, there is growing evidence for an ERα-independent mechanism promoting estrogen-induced breast cancer development (9). The estrogen metabolites such as 2,3-quinone catechols and 3,4-quinone catechols can induce the generation of reactive oxygen species (ROS), which, in turn, promote oxidative DNA base damage (3, 5). Estrogen metabolites can also directly induce genomic DNA damage (3, 5). The 3,4-quinones catechols can interact with adenine and guanine bases to form unstable depurinating 4-OH-E2/E1-1-N3 adenine and 4-OHE2/E1-1-N7 adducts. Spontaneous depurination of adducts can generate potentially mutagenic apurinic sites (also known as AP sites). Breast cancer patients or those at risk of developing breast cancer have been found to have higher levels of depurinating estrogen–DNA adducts in their urine compared with those not at increased risk for breast cancer development.

ROS generation and depurination induced by estrogen metabolites is an important source of DNA base damage, which is a strong stimulus for activation of DNA base excision repair (BER), a complex pathway that includes key enzymes, including AP endonuclease 1 (APE1), DNA polymerase β (pol β), flap endonuclease 1 (FEN1), poly (ADP-ribose) polymerase 1 (PARP1), X-ray cross-complementation group 1 protein (XRCC1), and DNA ligases (4). If BER is sub-optimal, DNA repair intermediates may be converted to single-strand breaks (SSB) and then to double-strand breaks (DSBs) during replication. DSBs activate key DNA damage sensing ataxia telangiectasia mutated (ATM), DNA-dependent protein kinase catalytic subunit (DNA-PKcs), and ataxia telangiectasia mutated and Rad3 related (ATR) protein kinases. Activated ATR and ATM phosphorylate Chk1 or Chk2, respectively; these, in turn, modulate a number of other proteins involved in DNA repair, cell cycle control, and apoptosis (7). Our hypothesis is that sub-optimal DNA repair may accelerate ROS-induced mutagenesis and lead to a mutator phenotype characterized by aggressive behavior in estrogen/ER-driven breast cancer. We investigated 1406 ER^+^ early-stage breast cancers with 20 years' long-term clinical follow-up data for pol β, FEN1, APE1, XRCC1, single-strand monofunctional uracil glycosylase-1 (SMUG1), PARP1, ATR, ATM, DNA-PKcs, Chk1, Chk2, p53, breast cancer susceptibility gene 1 (BRCA1), and topoisomerase 2 (TOPO2) expression.

Innovation The role of estrogen receptor (ER)-dependent cellular proliferation signaling is well established in breast cancer pathogenesis; however, very little is known about the clinical relevance of ER-independent mechanisms. Estrogen and estrogen metabolites induce reactive oxygen species generation that can promote oxidative DNA base damage which is repaired by base excision repair (BER). Impaired BER, therefore, could accelerate mutagenesis and promote a mutator phenotype in estrogen-driven breast cancers. The authors provide the first comprehensive clinical evidence that altered BER expression may be associated with aggressive estrogen-ER-driven breast cancers. Stratification by BER status may enable personalization of therapy in estrogen-driven breast cancers.

### Deregulated BER is independently associated with poor survival in ER^+^ breast cancers

The initial multivariate model included Pol β, FEN1, APE1, XRCC1, SMUG1, PARP1, ATR, ATM, CHK1, CHK2, p53, BRCA1, DNA-PKcs, TOPO2, lymph node status, and histological grade. Nonsignificant markers were then removed using a backward stepwise exclusion method until only significant markers remained. As shown in Table 1, low XRCC1 (*p*<0.01), low APE1 (*p*<0.01), low SMUG1 (*p*<0.01), and high FEN1 (*p*<0.01) remain independently associated with poor breast cancer-specific survival (BCSS). Lymph node stage (*p*<0.00001) and histological grade (*p*<0.00001) also remain independently associated with poor BCSS.

**Table 1.** Multivariate Cox Proportional Hazards Model in Estrogen Receptor-Positive Breast Cancers

*Variables*	*Beta*	p*-Value*	*Risk ratio*	*Risk ratio 95% lower*	*Risk ratio 95% upper*
XRCC1	−0.203954	**0.000219**	0.815500	0.731907	0.908640
FEN1	0.199727	**0.004731**	1.221069	1.063067	1.402556
SMUG1	−0.210251	**0.003919**	0.810381	0.702510	0.934816
APE1	−0.245472	**0.000754**	0.782335	0.678241	0.902406
Lymph node stage (continuous)	0.705445	**0.0000001**	2.024747	1.662938	2.465276
Histological grade	0.616616	**0.0000001**	1.852647	1.510526	2.272256

Bold values indicate significant *p*-values.

APE1, AP endonuclease 1; FEN1, flap endonuclease 1; SMUG1, single-strand monofunctional uracil glycosylase-1; XRCC1, X-ray cross-complementation group 1 protein.

### BER prognostic index stratifies patients into distinct prognostic groups in ER^+^ breast cancers

A BER prognostic index score (see Notes section) incorporating XRCC1, APE1, SMUG1, and FEN1 was developed and can be described by the following formula:

BER prognostic index score=XRCC1 (high: −0.20, low: 1)+FEN1 (high: 0.20, low: 1)+SMUG1 (high: −0.21, low: 1)+APE1 (high: −0.25, low: 1)

Sub-group 1 was defined as tumors with BER prognostic index score ranging from−0.46 to 0.34. Sub-group 2 was defined as tumors with BER prognostic index score ranging from 0.74 to 1.59. Sub-group 3 was defined as tumors with BER prognostic index score ranging from 1.95 to 2.80. Sub-group 4 was defined as tumors with a BER prognostic index score of more than 3.2.

**FIG. 1.**
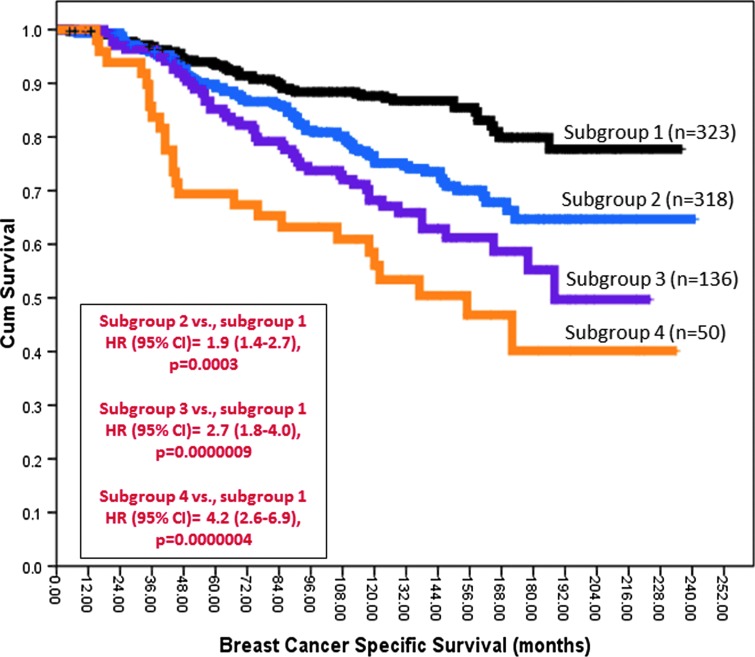
**BER and ER^+^ breast cancer.** Kaplan–Meier curves showing BCSS stratified based on BER prognostic index score. BCSS, breast cancer specific survival; BER, base excision repair; ER, estrogen receptor. To see this illustration in color, the reader is referred to the web version of this article at www.liebertpub.com/ars

**FIG. 2.**
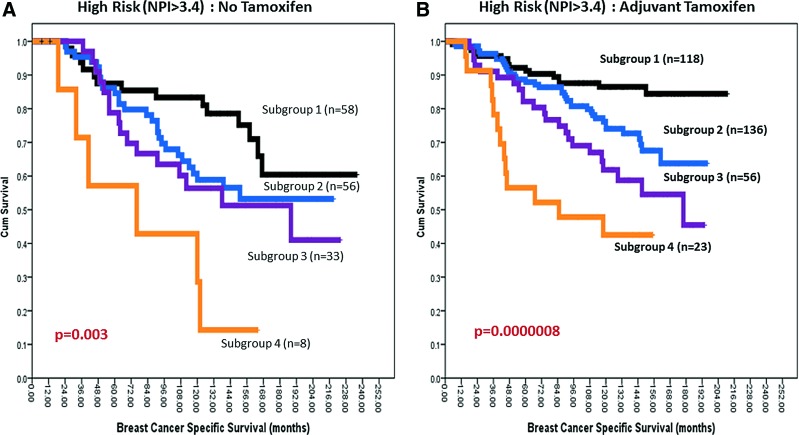
**Kaplan**–**Meier curves showing BCSS stratified based on BER prognostic index score in patients who did not receive tamoxifen (A) and who received adjuvant tamoxifen (B).** To see this illustration in color, the reader is referred to the web version of this article at www.liebertpub.com/ars

Survival analysis was conducted for the individual sub-groups. As shown in Figure 1, the BER prognostic index score stratified patients into four distinct sub-groups. Sub-group 1 had the best survival, whereas sub-group 4 had the worst survival (*p*<0.000001). The survival of patients in sub-group 2 (*p*<0.001) and sub-group 3 (*p*<0.000001) remains poor compared with sub-group 1. In patients who are at high risk (Nottingham index score >3.4) and did not receive adjuvant endocrine therapy, the survival of patients in sub-group 4 remains poor (Fig. 2A). Together, the data provides evidence that BER expression status may have prognostic significance in patients. We also evaluated whether BER had predictive significance in patients with ER^+^ tumours who received adjuvant tamoxifen therapy. As shown in Figure 2B, patients in sub-group 4 had the worst survival and patients in sub-groups 2 and 3 had intermediate prognosis compared with sub-group 1. These data suggest that BER expression may predict benefits from endocrine therapy.

### Deregulated BER is associated with aggressive ER^+^ breast cancers

The data presented earlier suggest that BER expression status may be a promising biomarker. To provide additional evidence that BER may impact aggressive biology, we conducted clinicopathological association studies in the various sub-groups. As shown in Table 2, larger size, higher grade, higher mitotic index, poor differentiation, high Ki67, Bcl-2 negativity, HER-2 overexpression, luminal B, and luminal B-Her 2 overexpression were more likely in sub-group 4, or 3 or 2 tumors when compared with tumors in sub-group 1 (*p*s<0.05). Interestingly, tubular, low grade, low mitotic index, and luminal A tumors were more common in sub-group 1 tumors.

**Table 2.** Clinicopathological Association in the Various Sub-Groups

*Factors*	*Expression*	*Sub-group 1* n *(%)*	*Sub-group 2* n *(%)*	*Sub-group 3* n *(%)*	*Sub-group 4* n *(%)*	p*-Value*
Lymph nodes						0.645
	0	201 (63)	194 (61)	72 (53)	33 (63)	
	1–3	98 (30)	104 (32)	51 (37)	15 (29)	
	>3	23 (7)	22 (7)	12 (10)	4 (8)	
Tumor size						**0.018**
	≤1 cm	44 (14)	38 (12)	12 (10)	4 (8)	
	>1–2 cm	18 (56)	155 (49)	62 (46)	25 (48)	
	>2–5 cm	95 (29)	118 (37)	59 (43)	21 (40)	
	>5 cm	2 (1)	8 (2)	3 (2)	3 (6)	
Grade						**0.000002**
	G1 (low)	87 (27)	58 (18)	23 (17)	9 (17)	
	G2 (intermediate)	152 (47)	136 (43)	45 (33)	15 (29)	
	G3 (High)	82 (26)	125 (39)	68 (50)	28 (54)	
Mitosis						**0.00008**
	M1 (low, <10)	178 (56)	129 (39)	46 (34)	19 (36)	
	M2 (medium, 10–18)	74 (23)	114 (36)	56 (42)	21 (40)	
	M3 (high, >18)	74 (23)	114 (36)	56 (42)	21 (40)	
Pleomorphism						**0.0005**
	Low	10 (3)	8 (2)	5 (4)	1 (2)	
	Moderate	185 (58)	161 (51)	49 (36)	20 (38)	
	High	126 (39)	148 (47)	81 (60)	31 (60)	
Differentiation						**0.017**
	Well differentiated	24 (8)	24 (8)	7 (5)	1 (2)	
	Moderate differentiated	144 (45)	106 (33)	48 (36)	16 (31)	
	Poor differentiated	153 (48)	188 (59)	80 (59)	35 (67)	
LVI	Presence	98 (30)	95 (30)	63 (46)	20 (39)	**0.003**
Tumor type						**0.006**
	Invasive ductal (NST)	130 (44)	149 (52)	72 (57)	26 (57)	
	Medullary	0 (0)	3 (1)	1 (1)	1 (2)	
	Tubular	91 (31)	61 (21)	37 (29)	5 (21)	
	Invasive lobular	44 (15)	39 (14)	11 (9)	6 (13)	
	Others	29 (10)	35 (12)	6 (5)	8 (17)	
Ki67	High expression	147 (53)	164 (60)	75 (65)	418 (59)	**0.012**
Bcl2	Negative expression	54 (18)	47 (16)	38 (30)	156 (20)	**0.0004**
HER2	Overexpression	13 (4)	17 (5)	14 (10)	1 (2)	**0.033**
Molecular sub-types						**0.014**
	Luminal A	153 (51)	132 (45)	53 (41)	14 (32)	
	Luminal B high Ki67	132 (44)	146 (49)	63 (48)	29 (66)	
	Luminal B-HER2 over expression	13 (5)	17 (6)	14 (11)	1 (2)	

Bold values indicate significant *p*-values.

NST, non-specific type.

## Concluding Remarks and Future Directions

Proficient DNA damage signaling and DNA repair machinery is critical for processing estrogen/estrogen metabolite-induced oxidative DNA base damage in cells (3, 5). In breast epithelial cells chronically exposed to estrogens, sub-optimal DNA repair and the resulting genomic instability may lead to accumulation of genetic mutations that eventually drive a cancerous phenotype. This is the first study that evaluates expression of BER in a large cohort of ERα-positive breast tumors. Here, we provide the first evidence that BER is deregulated in a proportion of ER^+^ tumors. We found impaired BER to be associated with aggressive phenotype and poor survival. We found that ER^+^ breast tumors could be categorized into four discrete subgroups based on BER protein levels. In addition to prognostic differences, significant differences in clinical characteristics were observed between subgroups. As expected, subgroups with poorer prognosis had significantly larger tumor size, higher grade, and other aggressive features. The clinicopathological associations shown here provide further evidence that BER deregulation may be involved in estrogen/ER-driven breast cancer pathogenesis and promote a mutator phenotype. However, detailed mechanistic studies in preclinical models will be required to confirm our hypothesis. A limitation to our study is retrospective in historical cohorts, albeit with 20 years' long-term follow-up data. Therefore, it would be essential to investigate the prognostic significance of BER in a more modern cohort where patients routinely receive adjuvant anthracycline-based chemotherapy schedules followed by aromatase inhibitor therapy in high-risk situations. However, it is important to note that in the group which received no adjuvant endocrine therapy, impaired BER remains associated with poor survival, suggesting that BER is an important prognostic biomarker. Interestingly, in patients who received tamoxifen, BER deregulation remains associated with poor survival, implying that BER may also have predictive significance in patients and the data suggest that additional therapy would be required to improve outcomes in such patients.

The mechanism for endocrine resistance in BER-impaired cells is largely unknown. However, recent studies suggest an interaction between BER and ER. For example, FEN1 has been shown to regulate ER-induced transcriptional response by enhancing the interaction of ER with estrogen response elements-containing DNA (6). Moreover, the mechanism of regulation of BER in ER^+^ tumours is also unknown. We have recently shown that FEN1 (2) may be regulated at the mRNA level; whereas for pol β levels, gene copy number changes as well as mRNA regulation may contribute to low expression (1).

PARP inhibitors that block single-strand break repair (SSBR), a pathway related to BER, have shown clinical benefit in BRCA1/2 germ line-deficient breast and ovarian cancers. Moreover, emerging evidence also suggests that triple negative breast cancers with BRCAness phenotype may also be suitable for synthetic lethality targeting using PARP inhibitors. The data presented in this study provide a tantalizing possibility for a similar synthetic lethality targeting in BER-deficient ER^+^ breast cancers using inhibitors of DSB repair pathway such as those targeting ATM, ATR, and DNA-PKcs that are currently undergoing pharmaceutical drug development. To provide evidence that such an approach is feasible, we have recently shown that XRCC1-deficient cancer cells are sensitive to ATM, DNA-PKcs, and ATR inhibitors (8).

In conclusion, BER status has prognostic and predictive significance in ER^+^ breast cancer patients. BER status-directed personalized therapy may be a promising approach in ER^+^ breast cancer.

## Notes

We investigated a consecutive series of 1406 patients with primary ERα positive invasive breast carcinomas who were diagnosed between 1986 and 1999 and entered into the Nottingham Tenovus Primary Breast Carcinoma series. All patients were treated uniformly in a single institution with a 20 year long-term clinical follow-up data and have previously been investigated in a wide range of biomarker studies (1, 2). The baseline demographics data have been summarized in previous publications (1, 2). Briefly, patients received standard surgery (mastectomy or wide local excision) with radiotherapy. Before 1989, patients did not receive systemic adjuvant treatment (AT). After 1989, AT was scheduled based on prognostic and predictive factor status, including Nottingham prognostic index (NPI), ERα status, and menopausal status. Patients with NPI scores of <3.4 (low risk) did not receive AT. In premenopausal patients with NPI scores of ≥3.4 (high risk), classical cyclophosphamide, methotrexate, and 5-flourouracil (CMF) chemotherapy was given; patients with ERα-positive tumors were also offered HT. Postmenopausal patients with NPI scores of ≥3.4 and ERα positivity were offered HT, while ERα-negative patients received classical CMF chemotherapy. The Reporting Recommendations for Tumor Marker Prognostic Studies (REMARK) criteria were followed throughout this study. This work was approved by Nottingham Research Ethics Committee. Tissue microarray (TMAs) were constructed and immunohistochemically profiled for Pol β, FEN1, APE1, XRCC1, SMUG1, PARP1, ATR, ATM, Chk1, Chk2, p53, BRCA1, DNA-PKcs, and TOPO2. Table 2 summarizes antigens, primary antibodies, clone, source, optimal dilution, and scoring system used for each immunohistochemical marker. We have reported the specificity of the antibodies used here in previous publications (1, 2). To validate the use of TMAs for immunophenotyping, full-face sections of 40 cases were stained and protein expression levels were compared. The concordance between TMAs and full-face sections was excellent (*k*=0.8). Positive and negative (by omission of the primary antibody and IgG-matched serum) controls were included in each run. Whole field inspection of the core was scored, and intensities of nuclear staining were grouped as follows: 0, no staining; 1, weak staining; 2, moderate staining; 3, strong staining. The percentage of each category was estimated (0%–100%). H-score (range 0–300) was calculated by multiplying intensity of staining and percentage staining. H-score cut-offs for individual markers are summarized in Table 3. Not all cores within the TMA were suitable for IHC analysis, as some cores were missing or lacked tumors. IHC data for all 14 DNA repair markers were available in 829 tumors. HER2 expression was assessed according to the new ASCO/CAP guidelines using chromogenic *in situ* hybridization. Data analysis was performed using SPSS (version 17; SPSS, Chicago, IL). Where appropriate, Pearson's χ^2^, Fisher's exact, χ^2^ for trend, Student's *t-*test, and ANOVAs one-way test were performed using SPSS software (version 17; SPSS). Multivariate analysis for survival was performed using the Cox hazard model. The proportional hazards assumption was tested using standard log-log plots. Hazard ratios and 95% confidence intervals (95% CIs) were estimated for each variable. All tests were two sided with a 95% CI. *p*-values for each test were adjusted with Benjamini and Hochberg multiple *p*-value adjustment, and an adjusted *p*-value of<0.05 was considered significant. DNA repair prognostic index was calculated from the β-values from the multivariate analysis of individual markers. A sum of “1” was assigned for low expression, and β-values were assigned for high expression. BER expression score for individual tumors was calculated as a sum of individual marker scores.

**Table 3.** Antigens, Primary Antibodies, Clone, Source, Optimal Dilution, and Scoring System Used for Each Immunohistochemical Marker

*Antigen*	*Antibody*	*Clone*	*Source*	*Antigen retrieval*	*Dilution/incubation time*	*Distribution*	*Scoring system*	*Cut-offs*
ER	Mouse MAb anti-ERα	SP1	Dako-Cytomation	Citrate pH6	1:150	Nuclear	Allred score	≥3 (Positive)
					30 min			
ER	Mouse MAb anti-ERα	EP1	Dako-Cytomation	Citrate pH6	1:80	Nuclear	% Positive cells	≥1% (Positive)
					30 min			
PR	Mouse MAb anti-PR	PgR636	Dako-Cytomation	Citrate pH6	1:125	Nuclear	% Positive cells	≥1% (Positive)
					30 min			
HER2	Rabbit antihuman c-erbB2	Polyclonal	Dako-Cytomation	None	1:400	Membrane	See text	See text
					60 min			
BRCA1	BRCA1	MS110	Calbiochem	Citrate pH6	1:100	Nuclear	% of positive cells	<25% (Negative)
					60 min			
ATM	Rabbit MAb anti-ATM	Y170	Abcam	Citrate pH6	1:100	Nuclear	% of positive cells	<25% (Negative)
					18 h			
ATR	Mouse MAb Anti-ATR	1E9	Novus Biologicals	Citrate pH6	1:20	Nuclear	H-score	≥60 (High)
					18 h			
Chk2	Rabbit polyclonal anti-Chk2	Ab47433	Abcam	Citrate pH6	1:100	Nuclear	H-score	≥100 (High)
					60 min			
pChk1	Rabbit anti-pChk1	Ab58567	Abcam	Citrate pH6	1:140	Nuclear	H-score	≥50 (High)
					60 min			
DNA-PKcs	Mouse MAb Anti-	3H6	Abcam	Citrate pH6	1:1000	Nuclear	H-score	>260 (High)
					20 min			
PARP1	Mouse MAb Anti-PARP1	7D3-6	BD Pharmingen	Citrate pH6	1:1000	Nuclear	% of positive cells	≥10% (Positive)
SMUG1	goat MAb anti-SMUG1		Acris Antibody GmbH	Citrate pH6	1/200	Nuclear	H-score	>35 (Positive)
					15 min			
APE1	Rabbit polyclonal anti-APE1	NB100-101	Novus Biologicals	Citrate pH6	1:500	Nuclear	H-score	≥100 (Positive)
					60 min			
XRCC1	Mouse MAb Anti-XRCC1	33-2-5	Thermo Scientific	Citrate pH6	1:200	Nuclear	% of positive cells	≥10% (Positive)
					20 min			
FEN1	rabbit polyclonal anti-FEN1	NBP1-67924	Novus Biologicals	Citrate pH6	1:200	Nuclear	H-score	≥100 (Positive)
					15 min			
TOP2A	Mouse MAb	KiS1	Dako-Cytomation	Citrate pH6	1:150	Nuclear/cytoplasmic	% of positive cells	>25% (Positive)
p53	Mouse MAb anti p53	DO7	Novocastra	Citrate pH6	1: 50	Nuclear	% of positive cells	≤20% (Negative)
					60 min			>20% (High)
Ki67	Mouse MAb anti-Ki-67	MIB1	Dako-Cytomation	Citrate pH6	1:300	Nuclear	% of positive cells	<10% (Low)
								10%–30% (Moderate)
								>30% (High)

ATM, ataxia telangiectasia mutated; ATR, ataxia telangiectasia mutated and Rad3 related; BRCA1, breast cancer susceptibility gene 1; DNA-PKcs, DNA-dependent protein kinase catalytic subunit; ER, estrogen receptor; PARP1, poly (ADP-ribose) polymerase 1.

## Authors' Contributions

S.M. and T.M.A.A.-F. designed and coordinated the study. P.M.M. performed immunohistochemical optimization and staining of markers. T.M.A.A.-F and A.A. conducted immunohistochemical scoring and analyses. T.M.A.A.-F., A.A., N.T., A.R.G., S.Y.T.C., and I.O.E. performed data interpretation. All authors were involved in writing, editing, and approval of this article.

## Acknowledgment

This work was supported by the Medical Research Council (CRTF) (grant number MR/J008001/1).

## Abbreviations Used

APE-1: AP endonuclease 1
ATM: ataxia telangiectasia mutated
ATR: ataxia telangiectasia mutated and Rad3 related
BCSS: breast cancer specific survival
BER: base excision repair
BRCA1: breast cancer susceptibility gene 1
CMF: cyclophosphamide, methotrexate, and 5-flourouracil 95%
CI: 95% confidence interval
DNA-PKcs: DNA-dependent protein kinase catalytic subunit
DSBs: double-strand breaks
ER: estrogen receptor
FEN1: flap endonuclease 1
NPI: Nottingham prognostic index
NST: non-specific type
PARP1: poly (ADP-ribose) polymerase 1
pol β: DNA polymerase β
ROS: reactive oxygen species
SMUG1: single-strand monofunctional uracil glycosylase-1
SSBR: single-strand break repair
SSB: single-strand breaks
TMA: tissue microarray
TOPO2: topoisomerase 2
XRCC1: X-ray cross-complementation group 1 protein
